# A Rare Presentation of Sjögren’s Syndrome With Hypokalemic Periodic Paralysis Treated Based on Renal Biopsy Findings

**DOI:** 10.7759/cureus.79404

**Published:** 2025-02-21

**Authors:** Masayuki Hara, Miki Ueda, Koichi Hata, Risa Ueno

**Affiliations:** 1 Department of Nephrology, Kyoto Saiseikai Hospital, Kyoto, JPN

**Keywords:** autoimmune disease, electrolyte imbalance, hypokalemia, periodic paralysis, renal biopsy, renal tubular acidosis, sjögren’s syndrome, steroid therapy, tubulointerstitial nephritis

## Abstract

Hypokalemic periodic paralysis (HPP) is a rare but significant manifestation of Sjögren’s syndrome, often associated with distal renal tubular acidosis (dRTA).

We report a case of a 60-year-old man with recurrent HPP due to severe hypokalemia. Despite the absence of sicca symptoms, serological testing and glandular function assessment confirmed the diagnosis of Sjögren’s syndrome. Laboratory findings revealed marked hypokalemia, metabolic acidosis, and excessive urinary potassium excretion, consistent with dRTA.

Renal biopsy showed a combination of hypokalemia-induced tubular damage and autoimmune tubulointerstitial nephritis (TIN). Potassium and bicarbonate supplementation improved symptoms initially, but persistent tubular dysfunction required a reassessment of the treatment strategy. Steroid therapy was introduced 14 months after renal biopsy due to ongoing tubular dysfunction, leading to significant renal function improvement and discontinuation of potassium supplementation.

This case highlights the importance of considering Sjögren’s syndrome in patients with unexplained hypokalemic paralysis, even in the absence of sicca symptoms. Furthermore, it underscores the potential role of renal biopsy in distinguishing hypokalemia-induced tubular dysfunction from autoimmune TIN, guiding treatment decisions in complex cases of Sjögren’s syndrome with renal involvement.

## Introduction

Hypokalemic periodic paralysis (HPP) is a condition characterized by episodic and intermittent muscle weakness and paralysis involving the limbs and trunk, associated with hypokalemia [1]. HPP can be classified as primary (hereditary) or secondary, with secondary forms resulting from underlying endocrine, renal, or autoimmune disorders. Diagnosis is based on medical history and clinical findings, and differential diagnosis is particularly crucial when secondary HPP is suspected. In such cases, treatment must address both the symptoms and the underlying primary condition [2]. Among the various etiologies, Sjögren’s syndrome is recognized as a rare cause of secondary HPP. In Sjögren's syndrome, hypokalemia is commonly observed as a result of distal renal tubular acidosis (dRTA), a frequent complication of the disease [3]. dRTA is characterized by impaired hydrogen ion secretion in the distal tubules, leading to chronic metabolic acidosis and compensatory urinary potassium loss. Although dRTA is often asymptomatic, severe cases can result in marked hypokalemia, occasionally leading to HPP. Rare cases of Sjögren's syndrome presenting with HPP have been reported [4,5].

The management of dRTA caused by Sjögren's syndrome is mainly symptomatic, focusing on correcting hypokalemia and acidosis through potassium and bicarbonate supplementation. However, the use of steroids or immunosuppressants for treating extraglandular manifestations remains controversial [3]. In particular, distinguishing between reversible tubular dysfunction due to chronic hypokalemia and autoimmune-mediated tubulointerstitial nephritis (TIN) is crucial for determining whether immunosuppressive therapy is warranted. Renal biopsy provides valuable pathological insights that may guide treatment decisions. This case report describes a rare instance of Sjögren's syndrome, in which HPP was the initial manifestation. It emphasizes the importance of considering Sjögren's syndrome in the differential diagnosis of HPP. Furthermore, renal biopsy played a pivotal role in providing insights that guided treatment decisions for this condition.

## Case presentation

A 60-year-old male patient presented to the emergency department with progressive weakness in both upper and lower extremities. Over the past year, he had intermittently experienced fatigue and muscle weakness following physical exertion. Ten days before admission, he played golf for three consecutive days, followed by three days of manual labor. Two days before admission, at approximately 10:00 PM, he began experiencing mild fatigue, which progressed to include weakness in all extremities the following day. On the day of admission, at around 02:00 AM, he attempted to go to the restroom but was unable to move, prompting him to seek emergency medical attention. The patient reported no dietary restrictions and maintained a normal diet. He denied any recent flu-like symptoms, fever, or diarrhea over the past month.

The patient had a history of urolithiasis, which was first diagnosed five years ago. He reported no family history of chronic diseases, no medication use, no history of alcohol consumption, and no known allergies. He had a history of smoking 10-20 cigarettes per day for 20 years, which he discontinued at the age of 40.

**Figure 1:**
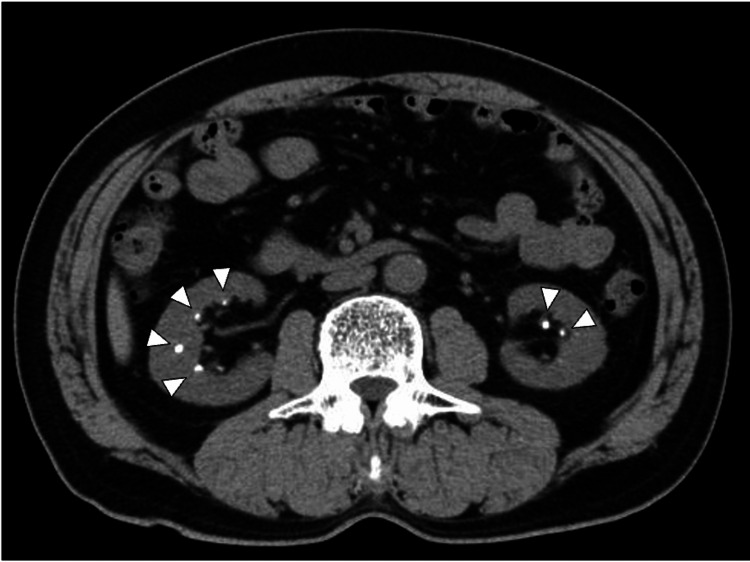
Abdominal computed tomography (CT) imaging showing bilateral renal calculi (arrows) without evidence of renal atrophy

On physical examination, the patient was alert and oriented. His height was 160 cm, weight 56.6 kg, and vital signs were stable, with a blood pressure of 125/77 mmHg, heart rate of 65 beats per minute, body temperature of 36.6°C, and oxygen saturation of 97% on room air. Cardiac and pulmonary auscultation revealed no abnormal findings. The abdomen was soft and non-tender, with no palpable masses. Neurologically, the patient could elevate his upper limbs and perform smooth distal movements, though grip strength was mildly reduced. He was unable to ambulate but could move his toes in the supine position, although raising his knees was not possible. The oral examination revealed recurrent dental caries, which the patient reported as a longstanding issue since childhood. Computed tomography (CT) imaging showed bilateral renal calculi without evidence of renal atrophy (Figure 1).

Laboratory tests showed marked hypokalemia, impaired renal function, normal anion gap metabolic acidosis, elevated tubular-interstitial markers, and proteinuria (Table 1).

**Table 1:** Summary of laboratory findings eGFR: estimated glomerular filtration rate; AST: aspartate aminotransferase; ALT: alanine aminotransferase; LDH: lactate dehydrogenase; TSH: thyroid-stimulating hormone; fT3: free triiodothyronine; fT4: free thyroxine; PRA: plasma renin activity; PAC: plasma aldosterone concentration; ACTH: adrenocorticotropic hormone; ANA: antinuclear antibody; sp gr: specific gravity; RBC sediment: red blood cell sediment; beta-2-MG: beta-2-microglobulin; NAG: N-acetyl-beta-D-glucosaminidase Urinary anion gap (UAG) and urinary potassium concentration vary depending on the patient's acid-base status and potassium balance, so their reference ranges are marked as "-".

Test	Value	Reference range
WBC	7360	3900-9800 /μL
Hemoglobin (Hb)	12.8	13.5-17.6 g/dL
Platelet	20.4 × 10⁴	13.0-36.9 /μL
Albumin	3.9	3.9-4.9 g/dL
Urea nitrogen	19	8-22 mg/dL
Creatinine	1.46	0.61-1.04 mg/dL
eGFR	38.2	> 0 mL/min/1.73 m²
Sodium	142	135-147 mEq/L
Potassium	1.7	3.6-5.0 mEq/L
Chloride	119	98-108 mEq/L
Calcium	9.6	8.6-10.1 mg/dL
Phosphorus	1.2	2.5-4.6 mg/dL
Magnesium	2.9	1.8-2.6 mg/dL
Total bilirubin	0.8	0.2-1.1 mg/dL
AST	31	10-40 IU/L
ALT	18	5-45 IU/L
LDH	172	115-245 IU/L
LDL cholesterol	103	70-139 mg/dL
HDL cholesterol	33	40-86 mg/dL
Triglycerides	96	35-149 mg/dL
Hemoglobin A1c	5.4	4.6-6.2%
C-reactive protein	0.4	< 0.3 mg/dL
TSH	0.325	0.61-4.23 μIU/mL
fT3	2.11	2.14-4.09 pg/mL
fT4	0.69	0.88-1.5 ng/mL
PRA	2.1	0.2-2.3 ng/mL/hr
PAC	55.9	4.0-82.1 pg/mL
ACTH	21.3	< 63.3 pg/mL
Cortisol	16	< 19.6 μg/mL
pH	7.29	7.35-7.45
pCO₂	31.1	35-45 mmHg
pO₂	61.7	75-100 mmHg
HCO₃	16	22-28 mmol/L
Lactate	15.7	< 2.0 mg/dL
Anion gap	7	8-12
IgG	2061	870-1700 mg/dL
IgA	480	110-410 mg/dL
IgM	32	33-190 mg/dL
ANA	x160	< x40
Anti-SSA-antibody	> 1200	< 10 U/mL
Anti-SSB-antibody	104	< 10 U/mL
sp gr	1.009	1.005-1.030
pH	7.0	5-8.0
Protein	1.15	< 0.11 g/g creatinine
Glucose	Negative	Negative
RBC sediment	< 1	< 5 /HPF
Urinary potassium	21.7	- mEq/g creatinine
Beta-2-MG	192307	< 300 µg/g creatinine
NAG	6.4	1.6-5.8 U/g creatinine
Urinary anion gap	6.9	-

These findings, including excessive urinary potassium excretion and the presence of renal calculi with a positive urinary anion gap, were consistent with a diagnosis of dRTA.

Further investigations revealed positive antinuclear antibodies (ANAs) and anti-SS-A antibodies, which, combined with findings from the Schirmer test, fluorescein staining, and gum test with a salivary flow rate reduced to 7.2 mL/10 minutes, supported the diagnosis of Sjögren’s syndrome. Additionally, a history of recurrent dental caries since childhood further suggested glandular involvement associated with the disease.

Following hospital admission, potassium supplementation resulted in significant improvement in muscle strength. However, urinary β2-microglobulin (β2MG) levels remained elevated, indicating persistent tubular-interstitial injury. Consequently, a renal biopsy was performed on the seventh day of hospitalization for further evaluation.

**Figure 2:**
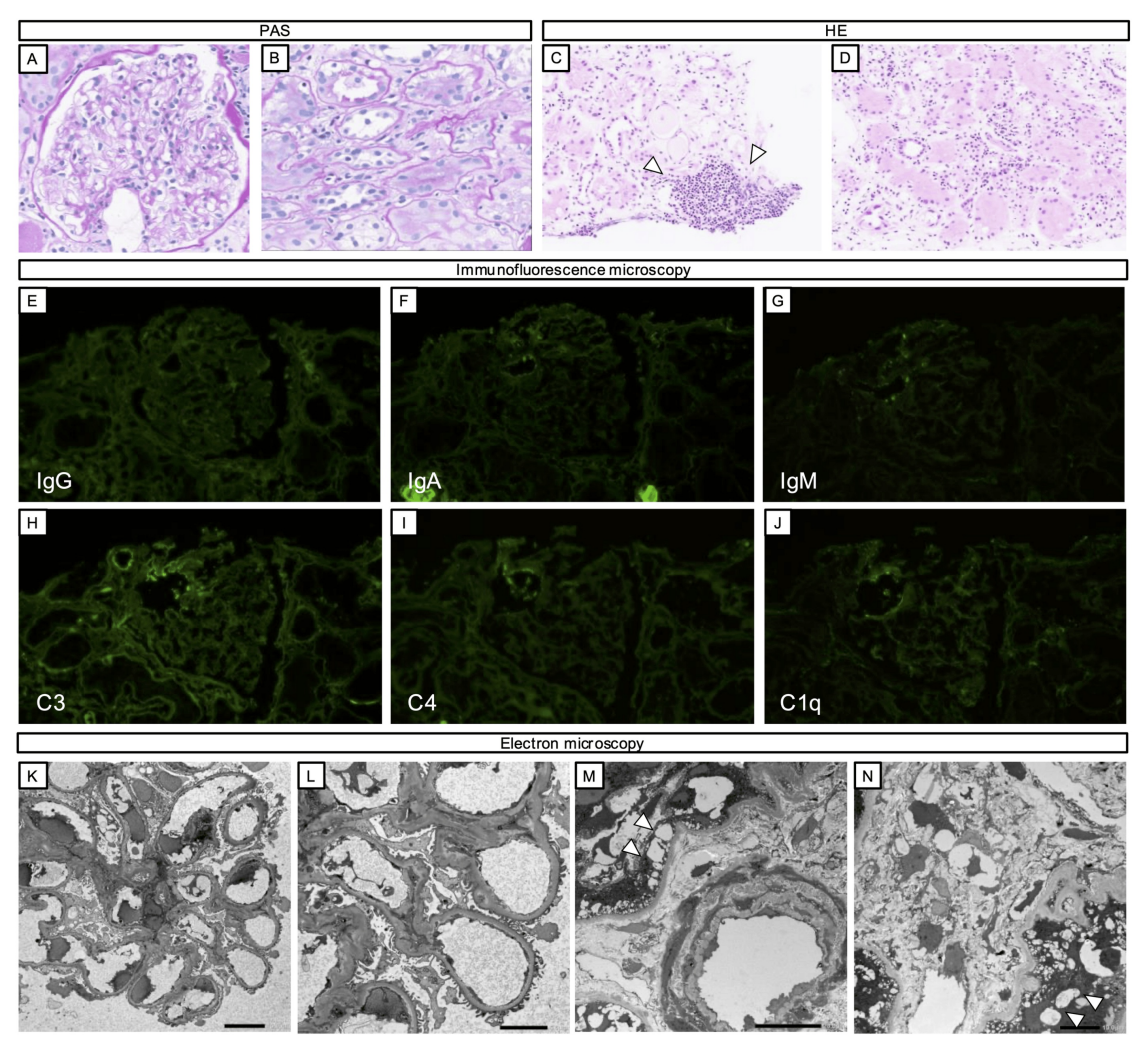
Renal histology (A, B) Light microscopy findings of periodic acid-Schiff (PAS) staining. (A) No significant pathological changes observed in the glomeruli. (B) Tubular epithelial cells exhibiting vacuolar degeneration. (C, D) Light microscopy findings of hematoxylin and eosin (H&E) staining. (C) Focal aggregation of lymphocytes and plasma cells observed in the interstitial area (arrows). (D) Mild diffuse interstitial nephritis around the tubules. (E-J) Immunofluorescence staining. No immune deposits detected by immunofluorescence staining. (K-N) Electron microscopy of the glomerulus (K, L) and tubules (M, N). (K, L) The glomerular basement membrane appears mostly uniform, with no evidence of lytic changes or fragmentation. No dense deposit is observed. (M, N) Vacuolar changes in the proximal tubular epithelium (arrows). Magnification: ×400 in (A, B, E-J, M), ×200 in (C, D), ×500 in (K, N), ×1200 in (L).

Light microscopy revealed no significant changes in the glomeruli. Vacuolar degeneration was noted in the tubular epithelial cells in the interstitial area. Lymphocytes and plasma cells were present around the tubules, but inflammatory cell aggregation was focal, and tubulitis was almost absent. Immunofluorescence staining showed no deposits of immunoglobulins or complements, and electron microscopy revealed no dense deposits (Figure 2).

**Figure 3:**
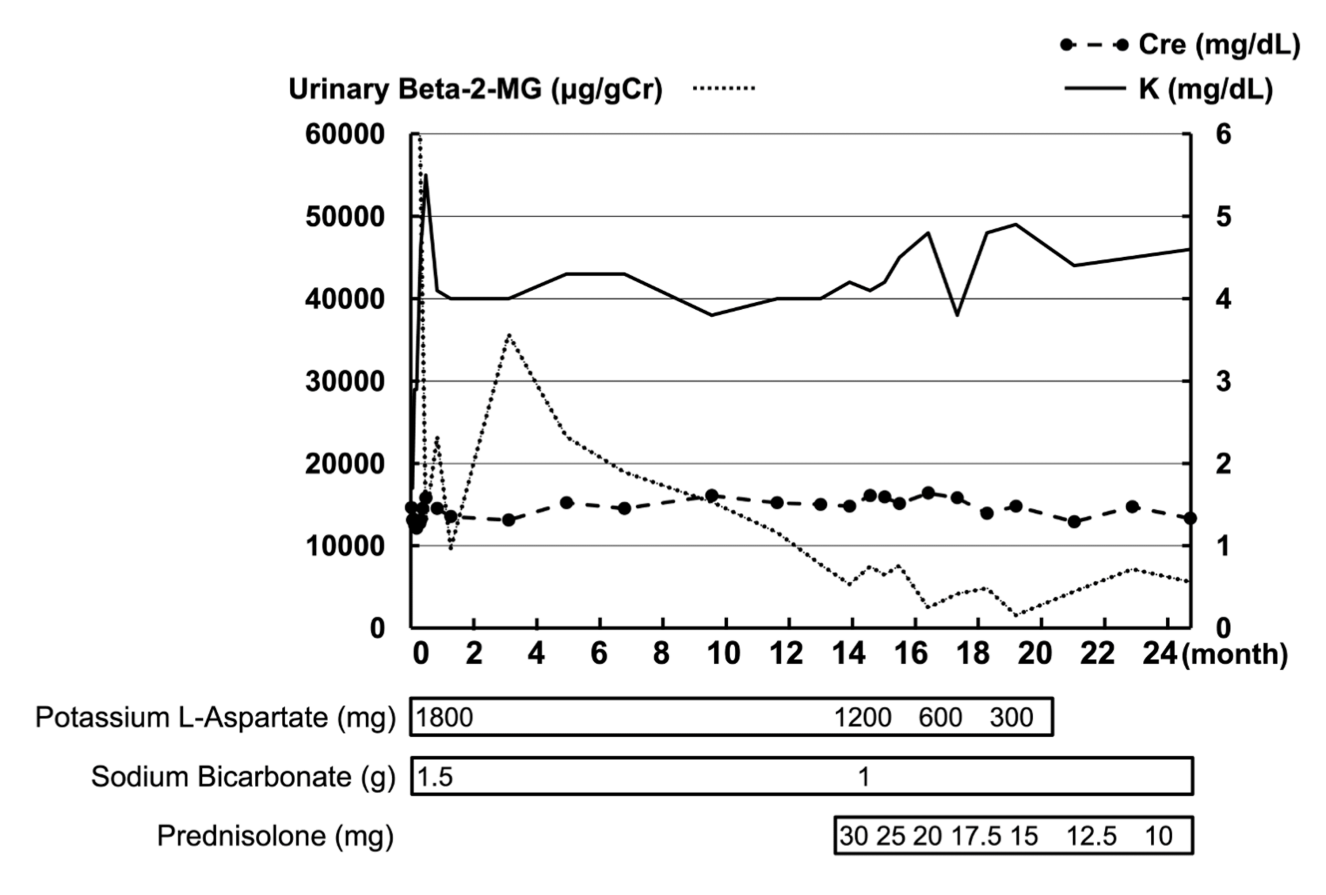
Clinical course 24 months after hospitalization

Based on these findings, the cause of the tubulointerstitial damage was considered to result from a combination of prolonged hypokalemia and Sjögren’s syndrome. Furthermore, the mild degree of peritubular cellular infiltration and the presence of vacuolar degeneration in the tubular epithelium suggested that hypokalemia-induced tubulointerstitial damage was the primary cause. Although potassium supplements and sodium bicarbonate were administered, β2MG levels did not normalize, and supplementation with potassium and sodium bicarbonate could not be discontinued (Figure 3).

Consequently, based on the renal biopsy findings, it was hypothesized that tubular damage was also influenced by inflammatory cell infiltration associated with Sjögren’s syndrome. Despite this hypothesis, steroid therapy was not initiated immediately after the biopsy due to the patient’s manageable condition with potassium and bicarbonate supplementation. Initially, conservative management was deemed appropriate, as electrolyte imbalances were controlled with supplementation. However, the persistent elevation of β2MG and the continued need for potassium and bicarbonate supplementation over the following months suggested an ongoing autoimmune process contributing to tubular dysfunction. Consequently, steroid therapy was initiated 14 months after the renal biopsy. Following treatment, urinary β2MG levels improved, and potassium supplementation was no longer required. The patient is currently undergoing gradual tapering of steroids in outpatient follow-up (Figure 3).

## Discussion

In this case, two important insights were gained regarding HPP. First, although Sjögren’s syndrome is rare, it should be recognized as a potential cause of severe hypokalemia leading to periodic paralysis, even in patients without typical sicca symptoms. This highlights the importance of including Sjögren’s syndrome in the differential diagnosis of unexplained hypokalemic paralysis. Second, while the management of dRTA in Sjögren’s syndrome is generally symptomatic, the findings from renal biopsy played a pivotal role in guiding the treatment strategy in this case. The biopsy provided evidence of autoimmune TIN, which justified the initiation of steroid therapy, resulting in significant clinical improvement.

Sjögren’s syndrome is a chronic autoimmune disease characterized by lymphocytic infiltration of exocrine glands, such as the salivary and lacrimal glands, due to immune system abnormalities. It predominantly affects middle-aged women (female-to-male ratio of 9:1), with an average age of 51.6 to 62 years [6]. The primary autoimmune symptoms include oral dryness caused by salivary gland dysfunction and eye dryness caused by lacrimal gland dysfunction, collectively referred to as sicca syndrome. Approximately 80% of patients present with sicca syndrome, while the remaining 20% are nearly asymptomatic [7]. In this case, the patient did not exhibit symptoms of sicca syndrome.

Additionally, about 15% of patients develop extraglandular symptoms affecting organs such as the lungs, muscles, joints, skin, nervous system, and kidneys, which contribute to higher mortality rates [3]. In our case, no extraglandular manifestations were observed apart from renal involvement. Renal involvement occurs in approximately 5% of patients with primary Sjögren’s syndrome, with 85% of these cases attributed to TIN and glomerulonephritis [3,8].

Hypokalemia caused by tubular dysfunction, such as renal tubular acidosis (RTA) or Fanconi syndrome, is a common finding in Sjögren’s syndrome and has been reported in 30-47% of patients with renal involvement. In this case, dRTA was observed. The precise mechanism by which Sjögren’s syndrome induces dRTA remains unclear, but autoimmune TIN is thought to be a contributing factor. Lymphocytes and plasma cells have been reported to infiltrate the renal interstitium and subsequently invade the tubular basement membrane and epithelial cells. This inflammatory process is believed to disrupt cellular structures, leading to dysfunction [4]. Immunocytochemical analysis of renal tissue in patients with Sjögren’s syndrome has demonstrated a loss of H-ATPase pumps, which are involved in proton secretion, in the cortical collecting ducts [9]. Additionally, the presence of autoantibodies against carbonic anhydrase II has been reported to inhibit its function, resulting in impaired H⁺ secretion [10].

Hypokalemia caused by RTA is often asymptomatic, and cases presenting with severe symptoms are rare [3]. However, in this case, the patient exhibited severe muscle weakness, particularly in the proximal muscles, associated with hypokalemia. The improvement in muscle strength following potassium supplementation confirmed that the weakness was attributable to hypokalemia. Additionally, blood and urine tests revealed that the cause of hypokalemia was dRTA. During potassium supplementation, urinary potassium excretion remained elevated, ranging from 30 to 50 mEq/gCr, even before serum potassium levels normalized. This persistent renal potassium wasting further supported this diagnosis. The patient also reported frequent episodes of muscle weakness and fatigue following physical exertion for approximately one year, leading to the diagnosis of HPP associated with Sjögren’s syndrome.

Severe hypokalemia leading to HPP is extremely rare in Sjögren’s syndrome [4,5,10,11]. Although the patient did not exhibit symptoms of sicca syndrome, the presence of hypokalemia and a history of recurrent dental caries since childhood raised suspicion of Sjögren’s syndrome. These findings ultimately led to the diagnosis. It is noteworthy that TIN associated with Sjögren’s syndrome can develop prior to the onset of sicca syndrome [12]. Therefore, it is crucial to consider Sjögren’s syndrome in the differential diagnosis of hypokalemia, even in the absence of typical sicca syndrome symptoms.

Renal biopsy is typically conducted to elucidate the underlying pathology of renal dysfunction and inform therapeutic decisions. However, in patients with Sjögren’s syndrome and hypokalemia, the underlying pathology is often presumed to be TIN, and renal biopsy is typically not performed [3].

In this patient, recurrent episodes of HPP suggested prolonged hypokalemia, prompting a renal biopsy to differentiate whether the abnormalities in tubular-interstitial markers were due to long-standing hypokalemia or Sjögren’s syndrome. Chronic hypokalemia has been reported to cause tubular damage, characterized by irregularly sized vacuoles in the tubular epithelium, particularly in the proximal tubules [13]. Histological features of Sjögren’s syndrome, on the other hand, include lymphocytic infiltration in the interstitial tissue, predominantly composed of CD4 T lymphocytes, along with interstitial fibrosis and tubular atrophy as the disease progresses [14].

In this case, renal biopsy revealed prominent vacuolar degeneration in the tubular epithelial cells, consistent with the effects of hypokalemia, while lymphocytic infiltration in the renal interstitium was relatively mild. Based on these findings, hypokalemia was considered the primary cause of tubular-interstitial damage, and conservative treatment with potassium supplementation was initiated. Muscle weakness improved rapidly, and post-exercise muscle weakness did not recur. However, despite normalized serum potassium levels for several months, tubular-interstitial markers did not improve, and the required dosage of potassium supplementation could not be reduced. These findings suggested that tubular dysfunction associated with Sjögren’s syndrome was also contributing to the condition. Importantly, renal biopsy allowed for the differentiation between hypokalemia-induced tubular damage and autoimmune-mediated TIN, providing critical insights for appropriate management.

The treatment of Sjögren’s syndrome primarily focuses on symptomatic management of sicca syndrome. The management of extraglandular manifestations, such as TIN and associated RTA, remains controversial. Standard therapy typically involves correcting acidosis and hypokalemia with bicarbonate and potassium supplements. While some reports have demonstrated the efficacy of steroids or immunosuppressive agents in certain cases [4,11], others have shown no clinical benefit from such therapies [12]. Previous reports of steroid ineffectiveness in Sjögren’s syndrome-associated dRTA may have involved cases with more advanced tubulointerstitial fibrosis or irreversible tubular damage. In contrast, our patient demonstrated relatively mild interstitial infiltration without severe fibrosis, which may have contributed to the observed steroid responsiveness.

In this patient, steroid therapy improved tubular-interstitial markers, allowing for the discontinuation of potassium and bicarbonate supplementation. This case highlights the potential utility of renal biopsy in differentiating between tubular dysfunction caused by prolonged hypokalemia and that associated with Sjögren’s syndrome. Further research is needed to identify which patients with Sjögren’s syndrome and renal complications may benefit from immunosuppressive therapies.

## Conclusions

In conclusion, Sjögren’s syndrome should be considered as a potential cause of severe hypokalemia, even in the absence of typical sicca symptoms. Furthermore, in cases of Sjögren’s syndrome with severe hypokalemia, renal biopsy may provide critical insights into the underlying pathology and guide appropriate treatment. Previous case reports have described variable responses to steroid therapy in Sjögren’s syndrome-associated dRTA, likely influenced by the degree of tubulointerstitial fibrosis and inflammation. Our case highlights that renal biopsy findings can help distinguish cases where immunosuppressive therapy is beneficial, particularly in patients with mild interstitial infiltration and moderate fibrosis. Future research is essential to refine therapeutic strategies for extraglandular manifestations of Sjögren’s syndrome, particularly those involving renal complications.
